# The association between long working hours and hearing impairment in noise unexposed workers: data from the 5th Korea National Health and Nutrition Examination Survey (KNHANES 2010–2012)

**DOI:** 10.1186/s40557-016-0140-1

**Published:** 2016-10-06

**Authors:** Jung-Woo Park, Jin-Soo Park, Seyoung Kim, Minkyu Park, Hyunrim Choi, Sinye Lim

**Affiliations:** 1 Department of Occupational and Environmental Medicine, Kyung Hee University Hospital, Seoul, South Korea; 2 Department of Occupational and Environmental Medicine, School of Medicine Kyung Hee University, Seoul, South Korea

**Keywords:** Long working hours, Hearing impairment, KNHANES

## Abstract

**Background:**

This study is aimed at finding out the relationship between long working hours, one of major job stress elements, and hearing impairment in unexposed workers to occupational and environmental noise.

**Methods:**

This study was performed on 1628 regular, full-time wage workers between the age of 25-64 who indicated in the survey of having no experience of exposure to noise, normal otoscopic findings, and not suffering from diabetes based on the data from the fifth Korea National Health and Nutrition Examination Survey (KNHANES 2010–2012). The average working hours per week was categorized into 40 h and lower group, more than 40 to 48 h group, more than 48 to 60 h group, and more than 60 h group. The groups were defined as suffering from low or high frequencies hearing impairment if the average hearing threshold for 0.5, 1, 2 kHz or 3, 4, 6 kHz in both ears exceeds 25 dB based on the pure tone audiometry. The association between average weekly working hours and hearing impairment was analyzed using logistic regression after gender stratification.

**Results:**

The prevalences of low and high frequencies hearing impairment in male workers were 4.3 and 28.6 %, respectively, which were much higher than female’s prevalence of 2.7 and 11.1 %. For male workers, no significant association was found between average weekly working hours and low and high frequencies hearing impairment. For female workers, odds ratios (OR) of low and high frequencies hearing impairment were 4.22 (95 % confidence interval (CI) 1.09–16.27) and 4.49 (95 % CI 1.73–11.67), respectively, after controlling for several related factors, such as, age, Body Mass Index (BMI), socio-economic status, health-related behavioral, and occupational characteristics variables, in the final model in the group working more than 60 h compared to the group working 40 h and lower. In addition, a dose-response relationship was observed that ORs of low and high frequencies hearing impairment were increased according to increasing average weekly working hours.

**Conclusions:**

The association between long working hours and hearing impairment in both low and high frequencies was significant in Korean female workers with a dose-response relationship. Therefore, the law to change the culture of long working hours should be enacted in order to protect the workers’ health and improve the quality of life in Korean workers.

## Background

The International Labor Organization (ILO) announced the first agreement limiting daily working hours to 8 h and weekly working hours to 48 h for the manufacturing sector in 1919. Around 100 years have passed since then, and the statutory working hours have decreased gradually with many countries currently limiting it to 40 h per week [1]. However, there is a difference in the actual working hours by country with many developing countries still showing long working hours compared to developed countries due to reasons of low hourly wage, non-existence of a labor union, and insufficient labor market management capacity [2]. In the case of Korea, despite the fact that it has been industrialized as a typical country which has East Asia’s distinctive ‘long working hour culture’ [2], the characteristic of long working hours still exists, and it held the second-highest record for long working hours among the Organisation for Economic Co-operation and Development (OECD) countries in 2014 [3].

The results of previous studies suggested that long working hours have had influence in various fields. In the short term, long working hours increase fatigue through growing job demands, interference with life outside work, and shortening sleep time and cause negative health behaviors, such as, smoking and alcohol abuse [4]. These results increase the occurrence of accidents in workplace [5], mental illnesses [6], and cerebro-cardiovascular diseases [7] on a personal level and lead to social losses, such as, declining productivity [8] and frequent absences [9] in the long term.

Meanwhile, hearing impairment creates problems, such as, difficulty in formation of relationships, social isolation, and limited career choices and it has become an important social problem that causes mental illness like depression [10]. Increasing age, history of ear diseases, diabetes, and smoking are common causes of hearing impairment which lead to various problems mentioned above in the general population while the major cause is exposure to occupational noise in the working population [11–13].

In addition, some studies presented the association between stress and hearing problems. In a study conducted on symphony orchestra musicians occupationally exposed to loud noise frequently, it has been observed that there is a significant correlation between stress-related symptoms and hearing problems, with most hearing problems starting off gradually after a stressful life event [14]. Meanwhile, another study analyzed the impact of stress on hearing in workers unexposed to noise. A study analyzing the Swedish Longitudinal Occupational Survey of Health (SLOSH) data in 2008 reported that increase in stress, such as, change of occupation or risk of dismissal raised the prevalence of hearing impairment or tinnitus [15]. A case-control study conducted in Germany showed that patients with sudden hearing impairment and acute tinnitus symptoms even without being exposed to loud noise had a significantly higher level of stress compared to the control group, suggesting that stress can be considered a significant risk factor in occurrence of these diseases [16]. Based on these previous studies, the idea that stress can cause or aggravate hearing impairment was proposed [17].

However, most previous studies did not differentiate hearing impairment and tinnitus or hyperacusis, based on a self-reported survey regarding hearing impairment, and not excluding well-known hearing impairment related factors. Therefore, this study is aimed at revealing the association between one of major stress elements, long working hours, and hearing impairment by using the data from the fifth Korea National Health and Nutrition Examination Survey (KNHANES) and analyzing the working hours which have not been presented as the cause of hearing impairment until now, although being one of the most important stress factors in the occupational health field [18].

## Methods

### Study participants

The KNHANES is a study conducted on Koreans nationwide by the Korea Centers for Disease Control and Prevention with the first survey conducted in 1998 and the sixth survey currently in progress. The data from the fifth KNHANES carried out from 2010 to 2012 was used in this study. The survey was constructed with independency and homogeneity through the introduction of the Rolling Sampling Survey in each survey year. In the fifth KNHANES, 192 sampling areas were chosen each year representing Koreans and all members of the 3800 households were surveyed, therefore, 11,400 households from a total of 576 areas surveyed in 3 years.

This study was performed on regular, full-time wage workers between the age of 25–64 among the total study participants of the fifth KNHANES. Among them, 1815 workers unexposed to noise who answered ‘no’ to ‘have experience using earphones at a noisy place’, ‘have experience being exposed to noise at workplace’, and ‘have experience being exposed to instantaneous noise’, and also have been diagnosed normal for both ears in otoscopic examination conducted by a otolaryngologist using 4 mm degree endoscope were chosen. The final study population was determined to 1628 study participants by excluding workers diagnosed with diabetes to rule out the impact of diabetes on hearing impairment [19, 20], soldiers with possible frequent exposure to firing noise, and participants with missing question entry in each variable.

### Long working hours

The International Labor Organization (ILO) stated that working more than 48 h a week is considered a major job stress and that the occurrence of cerebro-cardiovascular diseases are highly associated with working hours exceeding 60 h per week [21]. Therefore, it was advised to avoid working more than 48 h per week and best not to exceed 40 h per week. Many countries have already limited the statutory working hours to 40 h per week [1], while paragraph 1 of Article 50 of the Korean Labor Standards Act of Korea also states that working hours should not exceed 40 h per week excluding recess hours [22].

Based on these standards, this study made evaluations by using the value of the response to the question ‘how many hours do you work per week on average at work place including overtime and night overtime, excluding lunch hours?’ and classifying them into group working 40 h and lower, group working more than 40 to 48 h, group working more than 48 to 60 h, and group working more than 60 h.

### Hearing impairment

To evaluate the hearing level of the study participants, a pure tone audiometry at 0.5, 1, 2, 3, 4, 6 kHz was conducted on both ears through the Entomed SA 203 in a double wall audiology booth. World Health Organization (WHO) had defined normal hearing as the average result recording below 25 dB at 0.5, 1, 2, 4 kHz for the good-ear side [23], and it is widely used as the standard in judging daily life auditory abilities. However, using such definition to judge the degree of hearing impairment will rule out unilateral hearing impairment and lead to the emergence of the problem of not being able to find out the pattern of hearing impairment. To resolve this problem, some studies have defined hearing impairment with the average hearing threshold in both ears [24], and analyzed the pattern of low and high frequencies hearing impairment [25]. Based on this information, this study defines the binaural pure-tone average of both ears at 0.5, 1, 2 kHz for low frequency and the binaural pure-tone average at 3, 4, 6 kHz for high frequency. In addition, the binaural pure-tone average of both ears for low frequency or high frequency exceeding 25 dB was judged as having hearing impairment.

### Other variables

In this study, some related factors that can affect hearing impairment suggested by previous studies were included. Some studies showed relatively consistent results that cerebro-cardiovascular risk factors, such as obesity and smoking, have a negative effect on hearing [26, 27]. These results were considered to be due to the fact that cochlea was vulnerable to ischemic changes [28]. In a research relating to the socio-economic status, high prevalence of hearing impairment was observed in deprived families. This finding was explained by frequent prematurity and low birth weight in the lower socio-economic status [29], and a negative tendency for the noisy environment and a favor for wearing hearing protection aids in the higher socio-economic status individuals [30]. In addition, there was a limited evidence that healthy lifestyles, such as moderate exercise and moderate alcohol consumption, can help prevent hearing loss [31, 32].

The related factors were constructed as follows to consider socio-economic status, health-related behavior characteristics, and occupational characteristics of the study participants. The age was classified into units of 10 years with Body Mass Index (BMI) of less than 18.5 kg/m^2^ classified as underweight, BMI of 18.5–25 kg/m^2^ normal weight, and BMI of over 25 kg/m^2^ overweight. Socio-economic status included level of educational attainment, marital status, and household income. The level of educational attainment was classified into less than high school, graduated from high school, and graduated from junior college or higher educational level. The marital status was classified into single, married and living together, and others (separated, death of spouse, divorced). The household income was classified into four classes of low, middle-low, middle-high, and high in accordance with the monthly average equivalent household income (monthly household income divided by the square root of number of household members).

Health-related behavior characteristic variables include smoking, alcohol consumption, and exercise status. Smoking was classified into non-smoker for participants who have smoked less than 100 cigarettes in their lifetime, ex-smoker for those who smoked more than 100 cigarettes in their lifetime but currently not smoking, and current smoker for those currently smoking and have smoked more than 100 cigarettes in their lifetime. Alcohol consumption was classified into non-drinker for participants who did not drink at all in the past year, light drinker for participants drinking less than twice a week or drinking less than seven glasses per occasion for male (less than five glasses for female), and high-risk drinker for those drinking more than twice a week or drinking more than seven glasses per occasion for male (more than five glasses for female). The exercise status was classified into exercise group for participants having conducted intense physical activities more than 3 days a week and over 30 min per occasion resulting in experiencing body pain or breathing heavily than usual while those not applicable to such activities were classified as non-exercise group.

Occupational characteristic variables include job type and shift work status. The job type was classified mainly into non-manual jobs for ‘manager’, ‘specialist and related business employee’, ‘office worker’, and ‘service industry employee’ and manual jobs for ‘workers skilled in agriculture and fishery’, ‘technician or related skill employee’, ‘machine operating or assembly worker’, and ‘simple labor employee’. The shift work status was classified into day work group and shift work group for participants applicable to work pattern other than day shift (evening shift, night shift, day and night regular shift, 24-hour shift, split shift, irregular shift, and others).

### Statistical analyses

The fifth KNHANES is designed with all Koreans living in Korea as the target population and it is a complex sampling design data extracted after conducting the initial area-stratification and then the secondary stratification of households within the area. In this study, analysis was carried out considering weight, stratified variables, and cluster variables so that the sample represents the population and prevents biased outcomes.

To examine the general characteristics of the study population, the frequency and the average of each independent variable were presented through stratification by gender while chi-square tests were conducted to compare the distribution and t-tests were conducted to figure out significant mean difference. Taking into consideration previous study outcomes showing significant health impacts of long working hours on female workers [33–35], the association between the independent variables and hearing impairment was gender-stratified and then chi-square tests were conducted, and the results were presented by classifying it into low and high frequencies hearing impairment. To find out the degree of the association between long working hours and low and high frequencies hearing impairment by gender, logistic regression was applied to calculate odds ratio (OR). In the crude model, no adjustments were made while age, BMI, and socio-economic status variables were controlled in the second model, and health-related behavior characteristic variables and occupational characteristic variables were more controlled in the final model. SPSS v.19.0 was used for the statistical analyses and the significance level was set at *p* < 0.05.

## Results

### General characteristics of the study population

The general characteristics of the study population are presented in Table 1. Among all participants, male accounted for 53.7 % and female 47.3 %. The average age was 40.3 years for male and 39.6 years for female and the average age was slightly higher in male workers without statistical significance (*p* = 0.174). The proportion of educational attainment level of college or higher was significantly higher in male (*p* = 0.003) with the proportion of manual job in male at 31.0 % and higher than that of female of 16.8 % (*p* < 0.001). The proportion of shift work in female was slightly higher but not statistically significant (*p* = 0.239). The average working hours per week in male was 49.2 h, showing significantly longer than female’s 44.5 h (*p* < 0.001) while the percentage of group working more than 60 h in male was significantly higher than that in female, with male accounting for 12.6 % and female 7.2 % (*p* < 0.001).

**Table 1:** General characteristics of the study population

Variables	Total		Male		Female		*p*-value
	n^a^	n^b^(%^c^)	n^a^	n^b^(%^c^)	n^a^	n^b^(%^c^)	
Total	1628	3780449(100.0)	768	2030862(100.0)	860	1749588(100.0)	
Age (years)							
Mean ± SE	40.0 ± 0.3		40.3 ± 0.4		39.6 ± 0.4		0.174
25–34	444	1246970(33.0)	165	610588(30.1)	279	636382(36.4)	0.069
35–44	603	1368113(36.2)	311	791293(39.0)	292	576820(33.0)	
45–54	394	858442(22.7)	193	469842(23.1)	201	388600(22.2)	
55–64	187	306924(8.1)	99	159138(7.8)	88	147786(8.4)	
BMI (kg/m^2^)							
Underweight	72	153504(4.1)	8	20560(1.0)	64	132944(7.6)	<0.001
Normal	1092	2480000(65.6)	454	1190804(58.6)	638	1289196(73.7)	
Obese	464	1146946(30.3)	306	819498(40.4)	158	327448(18.7)	
Education							
≤ Middle school	170	362359(9.6)	58	151184(7.4)	112	211175(12.1)	0.003
High school	564	1427402(37.8)	252	725608(35.7)	312	701795(40.1)	
≥ College	894	1990688(52.7)	458	1154070(56.8)	436	836618(47.8)	
Marital status							
Never married	254	753440(19.9)	82	335275(16.5)	172	418165(23.9)	<0.001
Married	1289	2817011(74.5)	672	1653806(81.4)	617	1163205(66.5)	
Others	85	209998(5.6)	14	41780(2.1)	71	168218(9.6)	
Household income							
Low	56	162234(4.3)	19	73594(3.6)	37	88640(5.1)	0.017
Mid-low	381	1008432(26.7)	207	621443(30.6)	174	386988(22.1)	
Mid-high	576	1368482(36.2)	271	720932(35.5)	305	647550(37.0)	
High	615	1241302(32.8)	271	614892(30.3)	344	626410(35.8)	
Smoking							
Non-smoker	967	2044875(54.1)	181	478096(23.5)	786	1566780(89.6)	<0.001
Ex-smoker	267	638628(16.9)	236	570841(28.1)	31	67787(3.9)	
Current smoker	394	1096946(29.0)	351	981925(48.4)	43	115022(6.6)	
Alcohol							
None	269	549452(14.5)	80	212616(10.5)	189	336836(19.3)	<0.001
Light drinker	1123	2586279(68.4)	500	1290078(63.5)	623	1296201(74.1)	
Heavy drinker	236	644718(17.1)	188	528167(26.0)	48	116551(6.7)	
Exercise							
No	1335	3060829(81.0)	619	1602748(78.9)	716	1458080(83.3)	0.051
Yes	293	719621(19.0)	149	428113(21.1)	144	291507(16.7)	
Job type							
Non-manual	1278	2857188(75.6)	554	1401179(69.0)	724	1456009(83.2)	<0.001
Manual	350	923262(24.4)	214	629683(31.0)	136	293579(16.8)	
Shift work							
No	1373	3172611(83.9)	646	1730392(85.2)	727	1442219(82.4)	0.239
Yes	255	607838(16.1)	122	300469(14.8)	133	307369(17.6)	
Weekly working hours							
Mean ± SE	47.0 ± 0.3		49.2 ± 0.5		44.5 ± 0.5		<0.001
≤ 40	673	1460816(38.6)	243	625000(30.8)	430	835816(47.8)	<0.001
40 < x ≤ 48	401	930976(24.6)	176	480684(23.7)	225	450292(25.7)	
48 < x ≤ 60	399	1007298(26.6)	248	669145(32.9)	151	338153(19.3)	
> 60	155	381360(10.1)	101	256032(12.6)	54	125328(7.2)	

*SE* standard error

^a^unweighted count, ^b^estimated population size, ^c^column and estimated percentage

### Working hours by the characteristics of the study population

Average working hours per week according to the characteristics of study population are presented in Table 2. The largest percentage of the group working 40 h and lower was shown in the case of female, older age, higher educational level, high household income, non-smoker, non-drinker, non-manual job, and day work group. In contrast, the case of male, older age, lower educational level, manual job, and shift work showed the largest percentage of the group working more than 60 h. Notably, the oldest age group, 55–64 years, showed a bimodal distribution in working hours that the largest percentage was observed both in the group working 40 h and lower and more than 60 h. In addition, the proportion of current smoker and heavy drinker was the highest in the group working more than 60 h. There was no significant difference found in relation to BMI, marital status, and exercise.

**Table 2:** Baseline characteristics of study participants in relation to weekly working hours

Variables	Average working hours per week								*p*-value
	≤40		40 < x ≤ 48		48 < x ≤ 60		>60		
	n^a^	%^b^	n^a^	%^b^	n^a^	%^b^	n^a^	%^b^	
Sex									
Male	625000	30.8 %	480684	23.7 %	669145	32.9 %	256032	12.6 %	<0.001
Female	835816	47.8 %	450292	25.7 %	338153	19.3 %	125328	7.2 %	
Age (years)									
Mean ± SE	40.6 ± 0.4		39.1 ± 0.6		39.0 ± 0.6		42.3 ± 1.0		0.003
25–34	434679	34.9 %	352466	28.3 %	352545	28.3 %	107280	8.6 %	0.021
35–44	532509	38.9 %	306509	22.4 %	408269	29.8 %	120827	8.8 %	
45–54	353381	41.2 %	206971	24.1 %	196742	22.9 %	101348	11.8 %	
55–64	140247	45.7 %	65030	21.2 %	49742	16.2 %	51905	16.9 %	
BMI (kg/m^2^)									
Underweight	66465	43.3 %	28302	18.4 %	54891	35.8 %	3845	2.5 %	0.118
Normal	984258	39.7 %	589535	23.8 %	670664	27.0 %	235542	9.5 %	
Obese	410092	35.8 %	313138	27.3 %	281742	24.6 %	141973	12.4 %	
Education									
≤ Middle school	116233	32.1 %	77087	21.3 %	106274	29.3 %	62766	17.3 %	0.008
High school	530333	37.2 %	342979	24.0 %	375414	26.3 %	178677	12.5 %	
≥ College	814249	40.9 %	510911	25.7 %	525610	26.4 %	139917	7.0 %	
Marital status									
Never married	268863	35.7 %	216956	28.8 %	192447	25.5 %	75174	10.0 %	0.681
Married	1104046	39.2 %	655606	23.3 %	766179	27.2 %	291180	10.3 %	
Others	87907	41.9 %	58413	27.8 %	48671	23.2 %	15007	7.1 %	
Household income									
Low	51287	31.6 %	31609	19.5 %	64473	39.7 %	14865	9.2 %	0.001
Mid-low	313868	31.1 %	231306	22.9 %	315850	31.3 %	147408	14.6 %	
Mid-high	529608	38.7 %	334116	24.4 %	365995	26.7 %	138763	10.1 %	
High	566053	45.6 %	333944	26.9 %	260980	21.0 %	80325	6.5 %	
Smoking									
Non-smoker	947579	46.3 %	533403	26.1 %	411757	20.1 %	152136	7.4 %	<0.001
Ex-smoker	220452	34.5 %	144646	22.6 %	207975	32.6 %	65555	10.3 %	
Current smoker	292784	26.7 %	252927	23.1 %	387565	35.3 %	163670	14.9 %	
Alcohol									
None	244192	44.4 %	143659	26.1 %	107894	19.6 %	53707	9.8 %	0.003
Light drinker	1031733	39.9 %	634730	24.5 %	700198	27.1 %	219618	8.5 %	
Heavy drinker	184891	28.7 %	152587	23.7 %	199206	30.9 %	108035	16.8 %	
Exercise									
No	1190610	38.9 %	728900	23.8 %	823902	26.9 %	317417	10.4 %	0.611
Yes	270206	37.5 %	202076	28.1 %	183396	25.5 %	63943	8.9 %	
Job type									
Non-manual	1237357	43.3 %	695717	24.3 %	695575	24.3 %	228538	8.0 %	<0.001
Manual	223459	24.2 %	235258	25.5 %	311722	33.8 %	152822	16.6 %	
Shift work									
No	1248109	39.3 %	809858	25.5 %	867887	27.4 %	246757	7.8 %	<0.001
Yes	212706	35.0 %	121118	19.9 %	139411	22.9 %	134603	22.1 %	

*SE* standard error

^a^estimated population size, ^b^row and estimated percentage

### Hearing impairment and its association with relevant variables

The prevalences of low and high frequencies hearing impairment for each independent variable was presented after gender-stratified analysis in Tables 3 and 4. The prevalences of low and high frequencies hearing impairment in male were 4.3 and 28.6 %, respectively, which are much higher than female’s prevalence of 2.7 and 11.1 %.

**Table 3:** Characteristics by hearing impairment in male subjects

Variables	Low frequency hearing threshold				High frequency hearing threshold			
	Normal^a^	Impaired^a^	Rate^b^	*p*-value^c^	Normal^a^	Impaired^a^	Rate^b^	*p*-value^c^
Total	1943952	86909	4.3 %		1450702	580160	28.6 %	
Age								
25–34	601667	8921	1.5 %	0.003	536836	73752	12.1 %	< 0.001
35–44	767129	24164	3.1 %		639708	151585	19.2 %	
45–54	433876	35966	7.7 %		228438	241405	51.4 %	
55–64	141281	17857	11.2 %		45720	113418	71.3 %	
BMI (kg/m^2^)								
Underweight	20560	0	0.0 %	0.594	14802	5759	28.0 %	0.987
Normal	1132208	58595	4.9 %		853128	337675	28.4 %	
Obese	791184	28314	3.5 %		582771	236726	28.9 %	
Education								
≤ Middle school	138168	13016	8.6 %	0.069	76552	74632	49.4 %	< 0.001
High school	684015	41593	5.7 %		449823	275785	38.0 %	
≥ College	1121769	32300	2.8 %		924326	229743	19.9 %	
Marital status								
Never married	330788	4487	1.3 %	0.285	293642	41634	12.4 %	0.001
Married	1571384	82422	5.0 %		1137760	516046	31.2 %	
Others	41780	0	0.0 %		19300	22480	53.8 %	
Household income								
Low	72438	1156	1.6 %	0.868	41672	31921	43.4 %	0.503
Mid-low	591940	29504	4.7 %		434735	186708	30.0 %	
Mid-high	690088	30844	4.3 %		522569	198364	27.5 %	
High	589487	25406	4.1 %		451726	163166	26.5 %	
Smoking								
Non-smoker	464148	13948	2.9 %	0.391	373600	104496	21.9 %	0.085
Ex-smoker	535904	34937	6.1 %		380557	190284	33.3 %	
Current smoker	943901	38024	3.9 %		696544	285380	29.1 %	
Alcohol								
None	200508	12108	5.7 %	0.382	141176	71441	33.6 %	0.608
Light drinker	1246716	43362	3.4 %		933381	356697	27.6 %	
Heavy drinker	496727	31440	6.0 %		376145	152022	28.8 %	
Exercise								
No	1539723	63025	3.9 %	0.428	1173529	429219	26.8 %	0.081
Yes	404229	23884	5.6 %		277173	150940	35.3 %	
Occupation								
Non-manual	1356037	45141	3.2 %	0.060	1086389	314789	22.5 %	< 0.001
Manual	587915	41768	6.6 %		364312	265371	42.1 %	
Shift work								
No	1656145	74247	4.3 %	0.966	1268234	462159	26.7 %	0.012
Yes	287807	12662	4.2 %		182468	118001	39.3 %	
Weekly working hours								
≤ 40	600759	24241	3.9 %	0.740	463403	161597	25.9 %	0.226
40 < x ≤ 48	452960	27724	5.8 %		328092	152592	31.7 %	
48 < x ≤ 60	646323	22822	3.4 %		498059	171086	25.6 %	
> 60	243910	12122	4.7 %		161147	94885	37.1 %	

^a^estimated population size

^b^prevalence rate

^c^tested by chi-square test

**Table 4:** Characteristics by hearing impairment in female subjects

Variables	Low frequency hearing threshold				High frequency hearing threshold			
	Normal^a^	Impaired^a^	Rate^b^	*p*-value^c^	Normal^a^	Impaired^a^	Rate^b^	*p*-value^c^
Total	1702929	46659	2.7 %		1555827	193761	11.1 %	
Age								
25–34	630799	5583	0.9 %	< 0.001	631885	4497	0.7 %	< 0.001
35–44	566561	10259	1.8 %		542400	34420	6.0 %	
45–54	374768	13832	3.6 %		300648	87951	22.6 %	
55–64	130801	16985	11.5 %		80894	66892	45.3 %	
BMI (kg/m^2^)								
Underweight	129378	3565	2.7 %	0.119	126952	5992	4.5 %	0.292
Normal	1247967	41229	3.2 %		1134145	155051	12.0 %	
Obese	325584	1864	0.6 %		294730	32718	10.0 %	
Education								
≤ Middle school	188166	23009	10.9 %	< 0.001	138642	72534	34.3 %	< 0.001
High school	686690	15105	2.2 %		616569	85226	12.1 %	
≥ College	828073	8545	1.0 %		800616	36002	4.3 %	
Marital status								
Never married	414719	3446	0.8 %	0.125	416248	1916	0.5 %	< 0.001
Married	1123927	39277	3.4 %		1002594	160611	13.8 %	
Others	164283	3936	2.3 %		136984	31234	18.6 %	
Household income								
Low	86509	2131	2.4 %	0.985	76211	12429	14.0 %	0.901
Mid-low	376350	10639	2.7 %		340629	46359	12.0 %	
Mid-high	628839	18711	2.9 %		581604	65946	10.2 %	
High	611231	15178	2.4 %		557382	69027	11.0 %	
Smoking								
Non-smoker	1526154	40625	2.6 %	0.455	1399823	166956	10.7 %	0.482
Ex-smoker	67787	0	0.0 %		61194	6593	9.7 %	
Current smoker	108988	6033	5.2 %		94810	20212	17.6 %	
Alcohol								
None	320909	15928	4.7 %	0.190	283099	53737	16.0 %	0.044
Light drinker	1269704	26496	2.0 %		1159958	136243	10.5 %	
Heavy drinker	112316	4235	3.6 %		112770	3781	3.2 %	
Exercise								
No	1417873	40208	2.8 %	0.663	1293030	165051	11.3 %	0.664
Yes	285056	6451	2.2 %		262797	28710	9.8 %	
Occupation								
Non-manual	1432594	23414	1.6 %	< 0.001	1348346	107663	7.4 %	< 0.001
Manual	270335	23244	7.9 %		207481	86098	29.3 %	
Shift work								
No	1402762	39457	2.7 %	0.780	1301174	141045	9.8 %	0.041
Yes	300167	7202	2.3 %		254653	52716	17.2 %	
Weekly working hours								
≤ 40	822475	13340	1.6 %	0.014	773741	62075	7.4 %	< 0.001
40 < x ≤ 48	442782	7511	1.7 %		412838	37454	8.3 %	
48 < x ≤ 60	322857	15296	4.5 %		279331	58822	17.4 %	
> 60	114816	10512	8.4 %		89917	35410	28.3 %	

^a^estimated population size

^b^prevalence rate

^c^tested by chi-square test

The results of the analysis conducted on the prevalences of hearing impairment according to independent variables in male showed that the prevalences of low and high frequencies hearing impairment increased by age (low frequency: *p* = 0.003, high frequency: *p* < 0.001) while the prevalence of high frequency hearing impairment was particularly high for low educational attainment (*p* < 0.001), others (separated, death of spouse, divorced) marital status (*p* < 0.001), manual job (*p* < 0.001), and shift work (*p* = 0.012). The prevalences of hearing impairment by average weekly working hours showed no significant difference in both low and high frequencies (low frequency: *p* = 0.740, high frequency: *p* = 0.226).

The prevalences of hearing impairment in female in both low and high frequencies increased by age (low frequency: *p* < 0.001, high frequency: *p* < 0.001), low educational attainment (low frequency: *p* < 0.001, high frequency: *p* < 0,001), and manual job (low frequency: *p* < 0.001, high frequency: *p* < 0.001). The characteristics showing any association with hearing impairment in only high frequency were non-drinkers (*p* = 0.044), others (separated, death of spouse, divorced) marital status (*p* < 0.001), and shift work (*p* = 0.041). The prevalences of hearing impairment by average weekly working hours in both low and high frequencies increased significantly as the average weekly working hours increased (low frequency: *p* = 0.014, high frequency: *p* < 0.001).

### The association between working hours and hearing impairment

To find out the association between average weekly working hours and hearing impairment, logistic regression was applied based on the group working 40 h and lower after gender stratification and presented in Table 5. For male workers, ORs of low and high frequencies hearing impairment with increased working hours were not consistent and statistically insignificant in the crude model with no adjustments with related factors. On the other hand, in the final model after controlling for age, BMI, socio-economic status, health-related behavior characteristics, and occupational characteristic variables, the risk of low and high frequencies hearing impairment in the group working more than 40 to 48 h, group working more than 48 to 60 h, and group working more than 60 h increased compared to the group working 40 h and lower without statistical significance.

**Table 5:** Odds ratios (OR) and 95 % confidence intervals (CI) for hearing impairment by gender according to weekly working hours

Weekly working hours	Male		Female	
	Low frequency hearing impairment	High frequency hearing impairment	Low frequency hearing impairment	High frequency hearing impairment
Model 1^a^				
≤ 40	1.00	1.00	1.00	1.00
40 < x ≤ 48	1.52(0.52–4.39)	1.33(0.8–2.22)	1.05(0.35–3.14)	1.13(0.61–2.09)
48 < x ≤ 60	0.88(0.30–2.56)	0.99(0.59–1.64)	2.92(0.85–10.06)	2.62(1.34–5.15)
> 60	1.23(0.38–4.03)	1.69(0.96–2.97)	5.64(1.65–19.34)	4.91(2.06–11.68)
Model 2^b^				
≤ 40	1.00	1.00	1.00	1.00
40 < x ≤ 48	1.83(0.61–5.45)	1.77(0.99–3.18)	1.49(0.46–4.89)	1.44(0.74–2.78)
48 < x ≤ 60	1.12(0.40–3.13)	1.45(0.84–2.49)	3.55(0.84–14.95)	3.84(1.82–8.1)
> 60	1.13(0.36–3.55)	1.70(0.87–3.31)	4.24(1.05–17.14)	5.12(1.85–14.18)
Model 3^c^				
≤ 40	1.00	1.00	1.00	1.00
40 < x ≤ 48	1.79(0.65–4.98)	1.71(0.95–3.08)	1.22(0.36–4.13)	1.28(0.65–2.54)
48 < x ≤ 60	1.10(0.44–2.77)	1.34(0.77–2.33)	2.93(0.54–15.74)	3.53(1.57–7.95)
> 60	1.22(0.31–4.86)	1.61(0.80–3.22)	4.22(1.09–16.27)	4.49(1.73–11.67)

^a^Model 1 : crude model

^b^Model 2 : adjusted with age, BMI and socioeconomic status (education, marital status and household income)

^c^Model 3 : Model2 + health-related behavioral characteristics (smoking, alcohol intake and exercise) and occupational characteristics (job type, shift work)

For female workers, in the crude model without controlling for any related factors, ORs of both low and high frequencies hearing impairment showed a dose-response relationship with increasing average weekly working hours. In particular, OR of low frequency hearing impairment was significantly highest in the group working more than 60 h recording 5.64 (95 % confidence interval (CI) 1.65–19.34), while OR of high frequency hearing impairment for the group working more than 48 to 60 h was 2.62 (95 % CI 1.34–5.15) and that in the group working more than 60 h was 4.91 (95 % CI 2.06–11.68). Also, in the final model after controlling for all related factors, the dose-response relationship was maintained between average weekly working hours and hearing impairment and ORs of low and high frequencies hearing impairment in the group working more than 60 h was 4.22 (95 % CI 1.09–16.27) and 4.49 (95 % CI 1.73–11.67) with statistical significance.

## Discussion

This study explored the risk of hearing impairment resulting from long working hours unexposed to occupational and environmental noise in Korean full-time wage workers based on the data from the fifth KNHANES. The risk of hearing impairment in female workers increased significantly in low and high frequencies while there was no significant result in male workers. Besides, a dose-response relationship was observed between average weekly working hours and the risk of low and high frequencies hearing impairment based on the group working 40 h and lower in female workers. The risk of hearing impairment significantly increased especially when the average weekly working hours exceeded 60 h in low frequency and exceeded 48 h in high frequency.

In the previous studies, the prevalence of hearing impairment was low in female workers [11] because the male workers are considered to be exposed more to noise than female workers in a working environment [36]. However, in this study, we explored the prevalence of hearing impairment in workers unexposed to occupational and environmental noise and the results still showed a low prevalence rate in female workers (male 28.6 % vs female 11.1 % in high frequency). The hearing protection effect of estrogen has been presented as the mechanism explaining this result. A large cohort study showed that elderly women have better hearing than elderly men and the protection effect of estrogen could be confirmed through study outcomes showing increased occurrence of hearing impairment in women with Tuner’s syndrome which prevent the production of estrogen due to ovarian dysgenesis [37].

Although the prevalence of hearing impairment was low in female workers in this study, the association between long working hours and hearing impairment was significantly high in female workers. These results are consistent with the outcomes of previous studies showing that the negative impact on health of long working hours is greater for female than male workers [38]. The gender difference could be explained by the possibility that their working conditions are differ due to difference of positions and job roles, although their occupation and working hours are the same. Even if their working conditions are the same, women could be exposed to more stress because of lower aerobic capacity and muscle force, gender segregation or relatively low wages [39]. Moreover, even if female workers are exposed to the same stress level, the impacts may be different by gender. Such difference between genders is supported by the evidence showing that male released stress quickly after work whereas the stress level in female during work was maintained even after work [40], because women performed more unpaid work for family demands, such as, housework and childcare compared to men. According to the time spent in unpaid work announced by OECD in 2015, women had 1.96 times more work than men on the average in OECD member countries, but in Korea, the figure recorded 5.05 times showing the highest concentration rate of unpaid work in women among the OECD member countries [41]. Through such results, it can be suggested that the impact on health caused by chronic cumulative stress can be more negative when working long hours in both paid and unpaid works for Korean female workers.

The mechanisms of long working hours raising the risk of hearing impairment can be explained by several aspects. First suggesting mechanism is the activation of the sympathetic nervous system caused by long working hours, changing and damaging the function of the cochlear blood flow [42]. The cochlea is vulnerable to ischemic changes because blood flow is supplied solely by the labyrinthine artery without the collateral circulation [43]. The function of the cochlea can be damaged if such ischemic changes occur in the stria vascularis of the external wall which plays an important role in maintaining cochlear homeostasis [28]. Animal test results show that the cochlear perilymphatic PO_2_ drops an average of 40 % when the cochlear blood flow is reduced up to 35 % and a significant hearing impairment occurs [44]. Also, the spiral modular artery branching off from the labyrinthine artery which provides blood flow to the external wall of the cochlea is rich in myofibril and locates along the sympathetic nervous system. This indicates that various internal and external factors constrict the blood vessels through sympathetic nerves and may cause ischemic changes to the external wall of the cochlea [45]. Juhn et al. reported that regular epinephrine injections had an impact on the cochlear homeostasis in an animal test using chinchillas and hearing impairment got worse in proportion to the total administration period [46]. Also, Horner et al. reported that the level of temporary hearing impairment caused by noise was reduced when a guinea pig was exposed to noise after removing the superior cervical ganglion, one of the main sympathetic nerves [47]. Through these study results, it is suggested that excessive sympathetic nerve acceleration may bring about changes in the cochlear homeostasis through ischemic changes, causing hearing impairment. In addition, hearing impairment caused by such ischemic changes can get worse due to high metabolic activity of cochlear hair cells [43].

Another mechanism of long working hours causing hearing impairment can be explained through oxidative stress. Oxidative stress is an element which can create pathophysiology of hearing impairment, damage the DNA, and have a negative impact on hearing impairment by damaging the cochlear hair cells through protein and lipid degradation and increased apoptosis [48]. There is a possibility that such oxidative stress which triggers hearing impairment can also be produced by long working hours. In a study conducted on hospital workers comparing the level of oxidative stress in blood before and after long working hours, it was confirmed that the level of oxidative stress after work increased significantly with an explanation that oxidative stress increase can be caused through continuous physical activity, mental stress, and anxiety or drowsiness [49]. Based on these, oxidative stress produced by long working hours can be suggested as having an impact on hearing impairment.

On the other hand, some animal studies present the outcome that short-term stress can show a hearing protection effect based on the explanation that glucocorticoid is produced through hypothalamic-pituitary-adrenal axis (HPA axis) and the glucocorticoid receptor (GR) which becomes activated as a result of this will start working by using heat shock proteins and antioxidants as mediators [50]. In a recent study on stress, the active process of environmental adaptation in response to such stress was defined as allostasis and it was explained that allostatic load is produced during this process [51]. Although this allostatic load can protect the body temporarily and help in adaption, it has a chance of causing diseases as pathophysiological changes occur with long-term existence. Previous studies have already found that long hours of GR activation resulting from stress may bring about negative changes in the central nervous system, such as, the prefrontal cortex [52, 53] and this change in the central nervous system will disturb the central auditory processing and interpretation, causing a negative effect on hearing. Therefore, there is a limitation to prove the hearing protection effect of stress with just the studies analyzing the effects of short-term stress.

In this study, the association between long working hours and hearing impairment was observed significantly in low frequency as well as in high frequency. Considering that hearing impairment by the major causes, such as age increase and noise exposure, was mainly observed in high frequency, it can be suggested that another pathological mechanism of hearing impairment may exist which is different from the mechanism of hearing impairment resulting from age increase or noise exposure. In a previous study, authors classified presbycusis as sensory, neural, strial, and cochlear conductive types according to pathogenesis. They reported that strial type was characterized by flat loss on pure tone audiometry and associated with ischemic changes of stria vascularis supplying the cochlea [54]. Especially, because this strial capillary network exists abundantly at the base of the cochlea rather than the apex, the apex of cochlea is relatively vulnerable to ischemic changes than the base. Therefore, this distribution of blood flow results in hearing impairment caused by ischemic changes typically present not only in high frequency but also in low frequency [55]. This supports that well-known cerebro-cardiovascular risk factors, such as smoking and diabetes, may cause hearing impairment across the entire frequency [12]. Like the cerebro-cardiovascular risk factors, long working hours is also believed to bring about ischemic changes through hyperactivity of sympathetic nervous system, causing hearing impairment in low frequency as well as high frequency through a similar pathological mechanism.

Although this study presents that long working hours may cause hearing impairment, another Korean study analyzing the association between stress and hearing impairment reported that there was no significant difference in the level of stress and hearing [56]. However, the study population of this study was college students who visited the hospital for a physical examination, showing limitation in judging the effect of exposure to chronic stress in the working environment because there was the problem of the overall stress level of the study participants being low. On the other hand, a Swedish study analyzing the association among job stress, long-term stress, and hearing impairment through the SLOSH data presented that the hearing problem increased significantly with increased stress with a dose-response relationship and the result was consistent with this study [15].

As far as we know, this study is the first study to explore the association between long working hours and hearing impairment in noise unexposed workers. This study secures representativeness and credibility using the data from the fifth KNHANES, performing hearing test and interviewing by trained researchers. This study aimed to reveal the association between long working hours and hearing impairment after controlling several related factors, such as, age, BMI, socio-economic status, health-related behavioral, and occupational characteristics, excluding not only noise exposure in the working area but also the possibility of noise exposure in everyday life, such as, the use of earphones at noisy places. Nevertheless, this study had some limitations as follows. Firstly, there was the limitation of not being able to include the past work history of the study population. Secondly, information bias may exist because the study relied on the self-reported survey instead of not measuring the actual level of noise exposure. Furthermore, study participants could not recall the whole exposure history to noise in their lifetime. Therefore, there may be a possibility that the association between long working hours and hearing impairment in this study has been biased. Thirdly, this study did not include all of the factors that could lead to hearing impairment like the histories of congenital diseases, middle ear diseases and exposures to physical trauma, medication, and toxic substances because these information was not surveyed in the original dataset, KNHANES. Fourthly, this study could not confirm the causal relationship that long working hours may cause hearing impairment because this study has a cross-sectional design.

In order to overcome the limitations of this study, a cohort study can be performed on selected workers unexposed to occupational and environmental noise through not only thorough history taking of work history, past medical history, exposures to noise, physical trauma, medication, and toxic substances, but also noise measurement of the working places and other additional tests to exclude middle ear diseases.

## Conclusions

The association between long working hours and hearing impairment in both low and high frequencies was significant in Korean female workers with a dose-response relationship. The findings of this study may add some evidence to the body of knowledge of the risk of long working hours, one of well-known risk factors of health. Therefore, the law to change the culture of long working hours should be enacted in order to protect the workers’ health and improve the quality of life in Korean workers.

## Abbreviations

BMI: Body mass index
CI: Confidence interval
GR: Glucocorticoid receptor
HPA: Hypothalamic-pituitary-adrenal axis
ILO: International labor organization
KNHANES: Korea National Health and Nutrition Examination Survey
OECD: Organization for economic cooperation and development
OR: Odds ratio
SLOSH: Swedish longitudinal occupational survey of health
WHO: World Health Organization
